# Supplementary figures and images for: Landscape structure evolution and ecological risk evaluation of oasis desert cities: A case study of Tiemenguan city
Source: PLoS One. 2025 May 7;20(5):e0321762. doi: 10.1371/journal.pone.0321762 (PMC12057882; doi:10.1371/journal.pone.0321762)

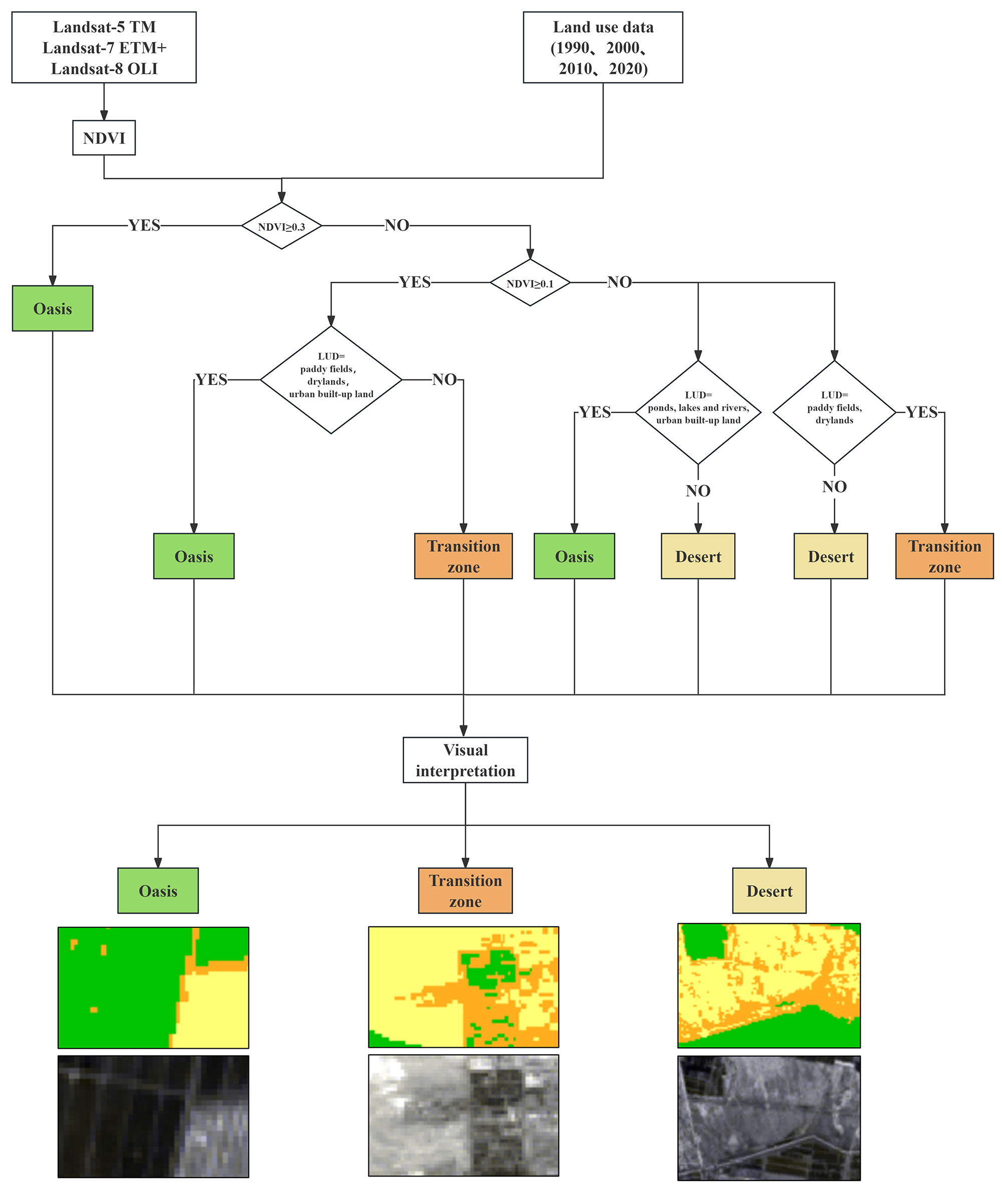

Supplement: S1 Fig — (TIF) [file pone.0321762.s001.tif]

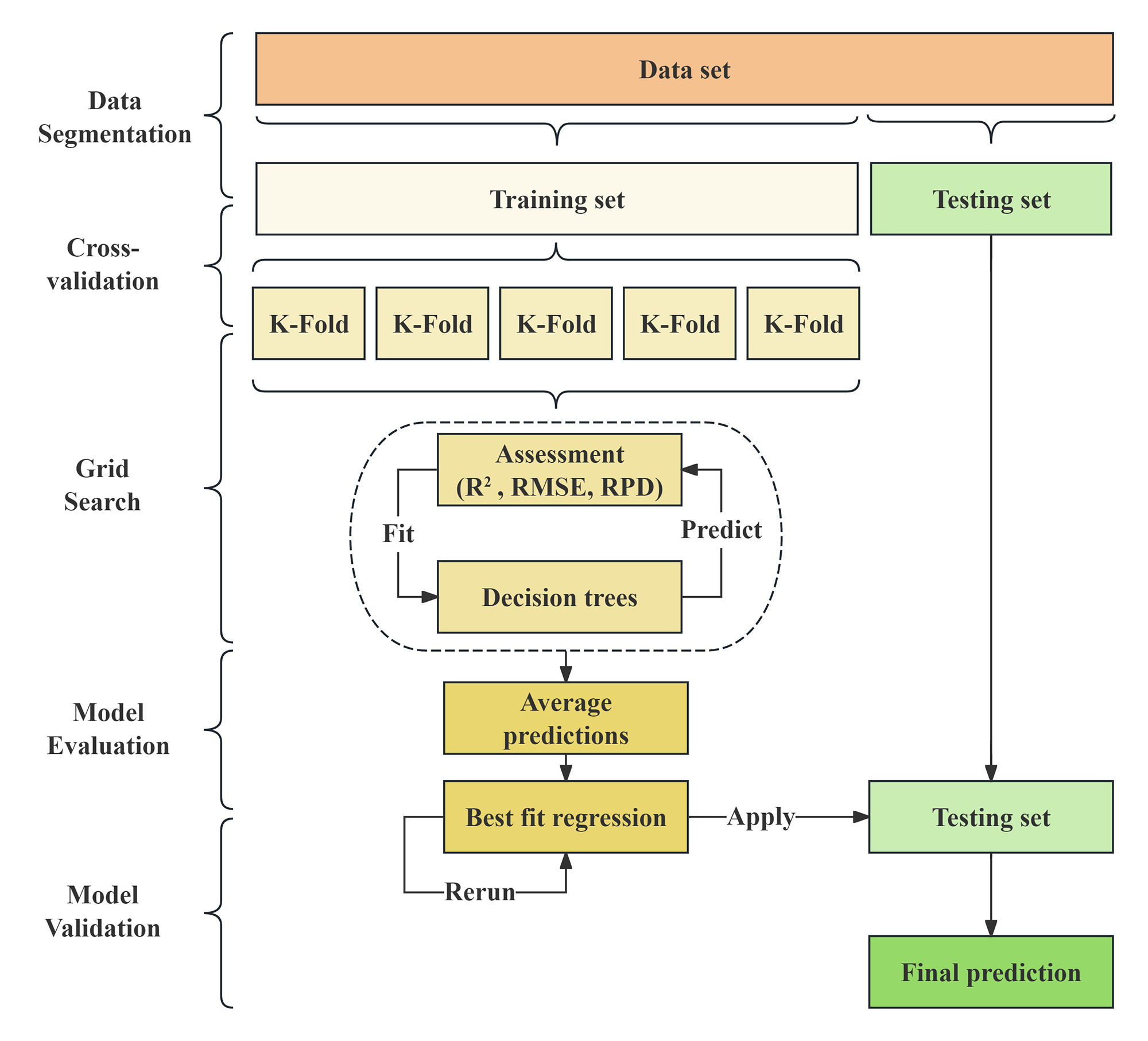

Supplement: S2 Fig — (TIF) [file pone.0321762.s002.tif]
