# Supplementary material for: Landscape structure evolution and ecological risk evaluation of oasis desert cities: A case study of Tiemenguan city
Source: PLoS One. 2025 May 7;20(5):e0321762. doi: 10.1371/journal.pone.0321762 (PMC12057882; doi:10.1371/journal.pone.0321762)
Supplement: Attachment — Submitted filename: Peer Review of Manuscript.pdf [file pone.0321762.s003.pdf]

Peer Review of Manuscript '*Landscape Structure Evolution and Ecological Risk Evaluation of Oasis Desert Cities: A Case Study of Tiemenguan City*'

1. Summary of the Research

This study investigates the spatiotemporal evolution of oasis landscape structures (OLS) and ecological risk indices (ERI) in Tiemenguan City, a typical oasis desert city in northwestern China, from 1990 to 2020. The research focuses on the relationship between OLS and ERI, the drivers of ecological risk, and projections under natural and government-regulated scenarios using models like Random Forest (RF) and PLUS. The main findings indicate continuous oasis expansion, decreasing ERI, and the dominance of socioeconomic factors in driving ecological risks. The study provides valuable insights into sustainable development in arid zones.

Strengths

- Comprehensive integration of remote sensing, modelling techniques, and ecological indices.
- Detailed methodology ensuring replicability and reliability.
- Novel insights into the dynamics of oasis desert cities under varying scenarios.

Weaknesses

- Limited generalizability due to the study's focus on a single city.
- Lack of clarity in certain figures and tables, which could hinder understanding.
- The discussion of limitations and future directions could be expanded.

Recommendation: Accept with minor revisions.

2. Examples and Evidence

Major Issues

1. **Clarity in Methodology**: While the methods are detailed, some equations (e.g., ERI calculation) lack clear explanations of parameters. Clarify how weights (a, b, c) were chosen and the rationale for using specific indices.
2. **Generalizability**: The focus on Tiemenguan City limits applicability to other arid zones. Future research should include comparative studies across multiple cities.
3. **Figures and Tables**: Certain visualizations (e.g., Fig. 6, Table 6) lack sufficient captions and are not self-explanatory. Ensure all visuals are clear and provide standalone explanations.
4. **Scenarios**: The assumptions for natural and government-controlled scenarios need better justification, including more details about specific policy interventions.

Minor Issues

1. Grammatical inconsistencies, particularly in the introduction and conclusion sections (e.g., sentence fragments in lines 90–92).
2. Enhance the discussion of RF model limitations and assumptions, particularly regarding socioeconomic data sources.
3. Standardize references to maintain consistency with journal formatting guidelines (e.g., line 477–501).
4. Expand the ethical implications of the study, especially given its focus on urban ecological sustainability.

3. Other Points (Optional)

Confidential Comments for the Editor

- The manuscript addresses a critical topic in urban sustainability and offers substantial contributions. However, the limited geographical scope and methodological ambiguities require attention before publication. The authors should prioritize enhancing clarity and rigour.
- I am available to review a revised version if necessary.
