# Supplementary material for: Landscape structure evolution and ecological risk evaluation of oasis desert cities: A case study of Tiemenguan city
Source: PLoS One. 2025 May 7;20(5):e0321762. doi: 10.1371/journal.pone.0321762 (PMC12057882; doi:10.1371/journal.pone.0321762)
Supplement: Attachment — Submitted filename: PONE-D-24-50511_reviewerENR.pdf [file pone.0321762.s004.pdf]

PLOS ONE

Landscape Structure Evolution and Ecological Risk Evaluation of Oasis Desert Cities: A Case study of Tiemenguan City

--Manuscript Draft--

**Manuscript Number:**	PONE-D-24-50511
**Article Type:**	Research Article
**Full Title:**	Landscape Structure Evolution and Ecological Risk Evaluation of Oasis Desert Cities: A Case study of Tiemenguan City
**Short Title:**	Landscape Structure Evolution and Ecological Risk Evaluation of Oasis Desert Cities
**Corresponding Author:**	Liu Hongguang<br>Shihezi University<br>shihezi, CHINA
**Keywords:**	Keywords：Evolution of oasis landscape structure；Ecological risk assessment；Driver analysis；Tiemenguan city；Multi-scenario forecasting
**Abstract:**	The rapid development of oasis desert cities adversely affects fragile ecosystems, preventing regional sustainable development. This study investigates the spatiotemporal evolution characteristics and potential quantitative relationship between oasis landscape structure (OLS) and the ecological risk index (ERI) and the growing trend in different development scenarios in Tiemenguan City, a typical oasis city in an arid zone in northwestern China, from 1990 to 2020. We calculated the ERI thresholds for different landscape types, classified different levels of ecological risk, and examined the factors influencing changes in ecological risk. The results showed the following. (1) The oasis area expanded continuously from 175.5 km2 to 345.3 km2 during 30 years. The transition and desert zones decreased by 49.7% and 37.9%, respectively. The ERI decreased and was strongly correlated with the OLS. The thresholds of the ERI in the oasis zone-transition zone and the transition zone-desert zone were 0.08-0.085 and 0.111-0.118, respectively. (2) Socioeconomic factors had the dominant influence on the spatiotemporal evolution of the ERI (the relative importance of infrastructure expansion, population density, and GDP exceeded 64%), and the influence of natural factors decreased over time. (3) The area of the Low-ERI region was 3.3% higher under the government control scenario than under the natural development scenario. The extent of the transition zone increased, and the growth rate of the oasis zone decreased over time in the former scenario. This study quantitatively evaluated each landscape type's ecological risk level and explored the effects of dynamic migration on the stability of their own and other landscapes. In addition, this paper provides a systematic research framework for ecological risk assessment of various landscape types in oasis-desert cities, and the results provide a certain scientific basis for ecological conservation and related research.
**Order of Authors:**	Sun Mingyue
	Liu Hongguang
	Dang Yingsheng
	Gong Ping
	Li Pengfei
	Fang Rui
	Cao Huan
	Li Xiang
	xia Hanji
	Ye Fuhai
	Guo yong
**Additional Information:**	

Powered by Editorial Manager® and ProduXion Manager® from Aries Systems Corporation

Question	Response
**Financial Disclosure**<br><br>Enter a financial disclosure statement that describes the sources of funding for the work included in this submission. Review the submission guidelines for detailed requirements. View published research articles from *PLOS ONE* for specific examples.<br><br>This statement is required for submission and **will appear in the published article** if the submission is accepted. Please make sure it is accurate.<br><br>**Funded studies**<br>Enter a statement with the following details:<br>• Initials of the authors who received each award<br>• Grant numbers awarded to each author<br>• The full name of each funder<br>• URL of each funder website<br>• Did the sponsors or funders play any role in the study design, data collection and analysis, decision to publish, or preparation of the manuscript?<br><br>Did you receive funding for this work?	Yes
Please add funding details.<br>as follow-up to "**Financial Disclosure**<br><br>Enter a financial disclosure statement that describes the sources of funding for the work included in this submission. Review the submission guidelines for detailed requirements. View published research articles from *PLOS ONE* for specific examples.<br><br>This statement is required for submission and **will appear in the published article** if the submission is accepted. Please make sure it is accurate.	This research study was supported by both Divisional and Municipal Science and Technology Tackling Program Projects (2024GG2301 and 2023GG1502).

**Funded studies**<br>Enter a statement with the following details:<br>• Initials of the authors who received each award<br>• Grant numbers awarded to each author<br>• The full name of each funder<br>• URL of each funder website<br>• Did the sponsors or funders play any role in the study design, data collection and analysis, decision to publish, or preparation of the manuscript?<br><br>Did you receive funding for this work?"	
Please select the country of your main research funder (please select carefully as in some cases this is used in fee calculation).<br>as follow-up to "**Financial Disclosure**<br><br>Enter a financial disclosure statement that describes the sources of funding for the work included in this submission. Review the submission guidelines for detailed requirements. View published research articles from *PLOS ONE* for specific examples.<br><br>This statement is required for submission and **will appear in the published article** if the submission is accepted. Please make sure it is accurate.<br><br>**Funded studies**<br>Enter a statement with the following details:<br>• Initials of the authors who received each award<br>• Grant numbers awarded to each author<br>• The full name of each funder<br>• URL of each funder website<br>• Did the sponsors or funders play any role in the study design, data collection and analysis, decision to publish, or preparation of the manuscript?<br><br>Did you receive funding for this work?"	CHINA - CN
**Competing Interests**	The authors have declared that no competing interests exist.

Use the instructions below to enter a competing interest statement for this submission. On behalf of all authors, disclose any competing interests that could be perceived to bias this work—acknowledging all financial support and any other relevant financial or non-financial competing interests.<br><br>This statement is required for submission and **will appear in the published article** if the submission is accepted. Please make sure it is accurate and that any funding sources listed in your Funding Information later in the submission form are also declared in your Financial Disclosure statement.<br><br>View published research articles from *PLOS ONE* for specific examples.<br><br>**NO authors have competing interests**<br><br>Enter: *The authors have declared that no competing interests exist.*<br><br>**Authors with competing interests**<br><br>Enter competing interest details beginning with this statement:<br><br>*I have read the journal's policy and the authors of this manuscript have the following competing interests: [insert competing interests here]*<br><br>* typeset	
**Ethics Statement**<br><br>Enter an ethics statement for this submission. This statement is required if the study involved:<br><br>• Human participants<br>• Human specimens or tissue<br>• Vertebrate animals or cephalopods<br>• Vertebrate embryos or tissues	N/A

• Field research<br><br>Write "N/A" if the submission does not require an ethics statement.<br><br>General guidance is provided below. Consult the submission guidelines for detailed instructions. **Make sure that all information entered here is included in the Methods section of the manuscript.**	

**Format for specific study types**<br><br>**Human Subject Research (involving human participants and/or tissue)**<br>• Give the name of the institutional review board or ethics committee that approved the study<br>• Include the approval number and/or a statement indicating approval of this research<br>• Indicate the form of consent obtained (written/oral) or the reason that consent was not obtained (e.g. the data were analyzed anonymously)<br><br>**Animal Research (involving vertebrate animals, embryos or tissues)**<br>• Provide the name of the Institutional Animal Care and Use Committee (IACUC) or other relevant ethics board that reviewed the study protocol, and indicate whether they approved this research or granted a formal waiver of ethical approval<br>• Include an approval number if one was obtained<br>• If the study involved *non-human primates*, add *additional details* about animal welfare and steps taken to ameliorate suffering<br>• If anesthesia, euthanasia, or any kind of animal sacrifice is part of the study, include briefly which substances and/or methods were applied<br><br>**Field Research**<br><br>Include the following details if this study involves the collection of plant, animal, or other materials from a natural setting:<br>• Field permit number<br>• Name of the institution or relevant body that granted permission	
**Data Availability**<br><br>Authors are required to make all data underlying the findings described fully available, without restriction, and from the time of publication. PLOS allows rare exceptions to address legal and ethical concerns. See the PLOS Data Policy and FAQ for detailed information.	Yes - all data are fully available without restriction

A Data Availability Statement describing where the data can be found is required at submission. Your answers to this question constitute the Data Availability Statement and **will be published in the article**, if accepted.<br><br>**Important:** Stating 'data available on request from the author' is not sufficient. If your data are only available upon request, select 'No' for the first question and explain your exceptional situation in the text box.<br><br>Do the authors confirm that all data underlying the findings described in their manuscript are fully available without restriction?	
**Describe where the data may be found in full sentences. If you are copying our sample text, replace any instances of XXX with the appropriate details.**<br><br>• If the data are **held or will be held in a public repository**, include URLs, accession numbers or DOIs. If this information will only be available after acceptance, indicate this by ticking the box below. For example: *All XXX files are available from the XXX database (accession number(s) XXX, XXX.).*<br>• If the data are all contained **within the manuscript and/or Supporting Information files**, enter the following: *All relevant data are within the manuscript and its Supporting Information files.*<br>• If neither of these applies but you are able to provide **details of access elsewhere**, with or without limitations, please do so. For example:<br><br>*Data cannot be shared publicly because of [XXX]. Data are available from the XXX Institutional Data Access / Ethics Committee (contact via XXX) for researchers who meet the criteria for access to confidential data.*<br><br>*The data underlying the results presented in the study are available from (include the name of the third party*	All relevant data are within the manuscript and its Supporting Information files.

and contact information or URL).<br>• This text is appropriate if the data are owned by a third party and authors do not have permission to share the data.<br><br>* typeset	
Additional data availability information:	Tick here if your circumstances are not covered by the questions above and you need the journal's help to make your data available.; Tick here if the URLs/accession numbers/DOIs will be available only after acceptance of the manuscript for publication so that we can ensure their inclusion before publication.

Powered by Editorial Manager® and ProduXion Manager® from Aries Systems Corporation

Landscape Structure Evolution and Ecological Risk Evaluation of Oasis Desert Cities: A Case study of Tiemenguan City

Mingyue Sun[1], Hongguang Liu[1*], Yingsheng Dang [1**], Ping Gong[1], Pengfei Li[1], Rui Fang[2], Huan Cao[2], Xiang Li[2], Hanji Xia[1], Fuhai Ye[1], Yong Guo[1]

[1] College of Water Conservancy & Architectural Engineering, Shihezi University, Shihezi 832000, People's Republic of China

[2] Hydrology and Water Resources Management Center of the Second Division of Xinjiang Production and Construction Corps, Tiemenguan 841007, People's Republic of China

* Corresponding author. College of Water Conservancy & Architectural Engineering, Shihezi

** Corresponding author.

E-mail: liuhongguang-521@163.com (H. Liu), 1075951141@qq.com (Y. Dang)

Abstract:

The rapid development of oasis desert cities adversely affects fragile ecosystems, preventing regional sustainable development. This study investigates the spatiotemporal evolution characteristics and potential quantitative relationship between oasis landscape structure (OLS) and the ecological risk index (ERI) and the growing trend in different development scenarios in Tiemenguan City, a typical oasis city in an arid zone in northwestern China, from 1990 to 2020. We calculated the ERI thresholds for different landscape types, classified different levels of ecological risk, and examined the factors influencing changes in ecological risk. The results showed the following. (1) The oasis area expanded continuously from 175.5 km^2 to 345.3 km^2 during 30 years.

The transition and desert zones decreased by 49.7% and 37.9%, respectively. The ERI decreased and was strongly correlated with the OLS. The thresholds of the ERI in the oasis zone-transition zone and the transition zone-desert zone were 0.08-0.085 and 0.111-0.118, respectively. (2) Socioeconomic factors had the dominant influence on the spatiotemporal evolution of the ERI (the relative importance of infrastructure expansion, population density, and GDP exceeded 64%), and the influence of natural factors decreased over time. (3) The area of the Low-ERI region was 3.3% higher under the government control scenario than under the natural development scenario. The extent of the transition zone increased, and the growth rate of the oasis zone decreased over time in the former scenario. This study quantitatively evaluated each landscape type's ecological risk level and explored the effects of dynamic migration on the stability of their own and other landscapes. In addition, this paper provides a systematic research framework for ecological risk assessment of various landscape types in oasis-desert cities, and the results provide a certain scientific basis for ecological conservation and related research.

Keywords：Evolution of oasis landscape structure；Ecological risk assessment；Driver analysis；Tiemenguan city；Multi-scenario forecasting

1. Introduction

Oases are crucial in ecological conservation and agricultural production in arid zones. They typically cover less than 10 percent of the region’s area but support more than 90 percent of the population [1]. The development of oases depends on water and heat conditions. These areas generally have an ecological gradient, i.e., the vegetation cover, biodiversity, and other characteristics change significantly from the desert to the oasis areas. The vegetation zone that

separates the desert zone from the oasis zone represents a transitional buffer (i.e., it captures wind-driven sand, serves as a soil and water conservation area, and ensures species diversity). The landscape is comprised of an oasis area, a transition zone, and a desert area from the inside to the outside [2]. A combination of factors such as climate change and human activities influences the evolution of this structure. On the one hand, rational land development and water management have accelerated the expansion of oasis zones [3]. However, wind erosion and water shortages adversely affect newly reclaimed areas, reducing the region's ecological stability, preventing oasis expansion, and potentially causing ecosystem degradation and desertification in local areas [4].

Analyzing changes in the oasis landscape structure (OLS) is significant for maintaining regional ecological stability and sustainable socioeconomic development. It is a hot research topic in arid and semi-arid regions [5]. Spectral index thresholding has been widely applied to relevant studies. The Normalized Difference Vegetation Index (NDVI) is often used to detect differences in vegetation cover over time and to analyze landscape change. However, the thresholds depend on the region and the remote sensing data source. For example, Ding et al. used NDVI values to delineate different zones. They defined areas with NDVI values of less than 15% as desert zones, those with values from 15%-30% as transition zones, and those with NDVI values $\geq$ 30% as oasis zones[6]. A study of oasis cities in the middle part of the Tarim River found that the average vegetation cover in the oasis zone-transition zone was 24%[7]. Zhang et al. set the NDVI thresholds at 20% (oasis zone-transition zone) and 35% (transition zone-desert zone). A study of oasis desert zones in the Hexi Corridor obtained an overall accuracy (OA) of the delineation results of more than 80% [8]. Therefore, choosing NDVI thresholds suitable for regional characteristics is critical to ensure high accuracy in classifying oasis landscape zones.

Ecological risk refers to potential damage to ecosystems, such as land degradation, landscape fragmentation, and biodiversity loss [9-11]. Multiple landscape ecosystems, such as mountains [12], lochs [13], oases [14], and cities [15] , have been studied. The geographical characteristics of the oasis region, such as fragile ecosystems and low resilience, have increased the difficulty and urgency of ecological risk management in the region. Many studies have used multiple ecological risk indicators (ERIs), including the ecological carrying capacity index [16] , the ecological vulnerability index [17] , the landscape disturbance index (LDI) [18] , and the degree of landscape ecological loss [19], to establish comprehensive and precise ecological risk evaluation frameworks. Due to the complex mechanisms of natural and socioeconomic factors and difficulties in data acquisition, many methods have been used to analyze complex interactions between factors and ERIs, such as geographically weighted regression [20] , Random Forest (RF) regression [21] , structural equation modeling [22] , and spatial error modeling[8]. It is crucial to incorporate regional characteristics and use multiple indicators and advanced analytical methods in analyzing the ecological risk of oases areas.

Simulating different future scenarios is necessary to evaluate risk. Model simulations have been used to predict future development and the dynamic evolution of oases. The Future Land Use Simulation (FLUS) model [23], Patch-generating Land Use Simulation (PLUS) model[24], Conversion of Land Use and its Effects at Small regional extent (CLUE-S) model [25] , and the Cellular Automata (CA)-Markov model[26] have been widely used. The applicability of a model depends on its ability to deal with spatial heterogeneity, data requirements, and ecosystem complexity. Suitable models must be chosen based on the research objectives and data, and their strengths and weaknesses should be considered to obtain an accurate prediction of the development

of oasis regions.

Xinjiang is located in an arid region. Tiemenguan City is a typical oasis city in Xinjiang. It has maintained a high level of economic development due to policies such as the Western Development Program. However, it suffers from water scarcity, and the ecosystem is unstable, adversely affecting human well-being [27, 28]. This study examines the correlation between the OLS and ERI in oasis areas. We assess the spatiotemporal characteristics of OLS and ERI and the impact of dynamic migration in the oasis area on the ecological stability of the transition zone and the desert area to determine whether the degradation of the transition zone increases the ecological risk of the oasis area. An ERI quantifies the potential risks associated with changes in landscape patterns to provide risk warnings for urban developme[illegible]he objectives of this study are to (1) analyze the potential correlation between the spatiotemporal characteristics of OLS and ERI from 1990 to 2020, (2) assess the relative importance of drivers of spatiotemporal changes using the ERI and the RF regression model, and (3) predict trends in the ERI and OLS for different land use scenarios from 2020 to 2030 using the PLUS model. Figure 1 shows the research framework.

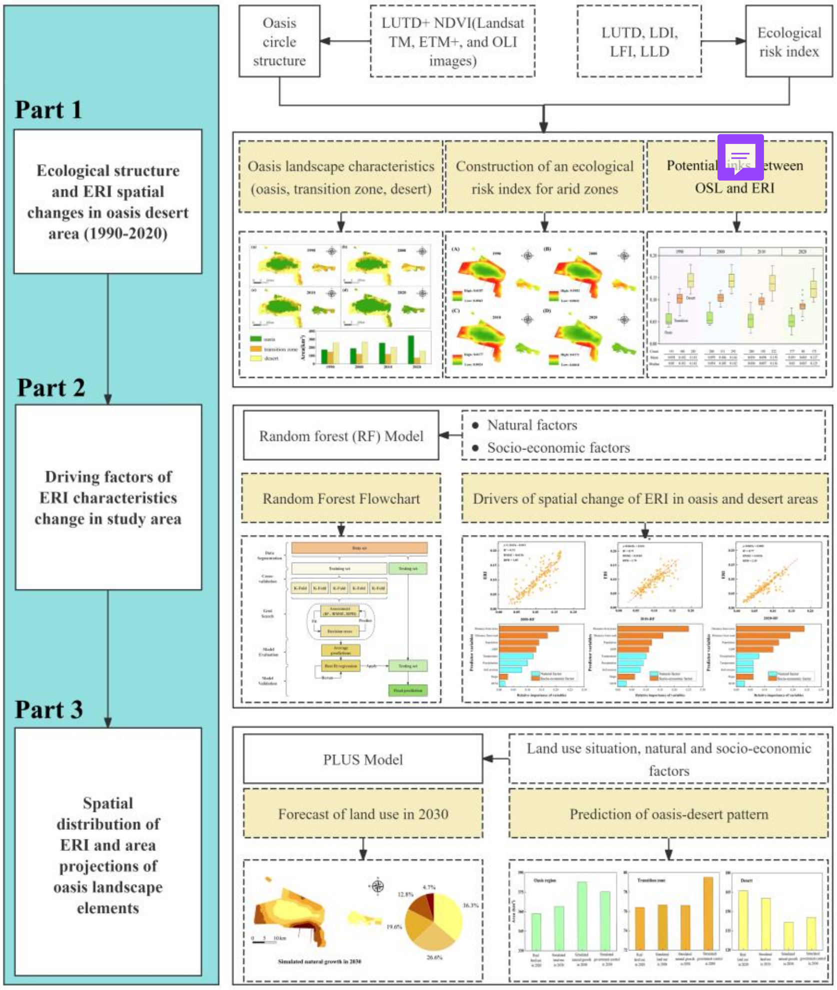

Fig. 1. Research framework.

Note: LUTD, LDI, LFI, and LLD stand for Land Use Type Data, Landscape Disturbance Index, Landscape Fragility Index, and Landscape Loss Index, respectively.

2. Materials and methods

2.1. Study area

Tiemenguan City is located at the intersection of the southern foothills of the Tianshan Mountains and the northern slopes of the Kunlun Mountains (Fig. 2). It is located on an important route in the middle section of the ancient Silk Road (41°45′–41°49′N, 85°53′–86°05′E). The area is located on the Eurasian continent, far away from the sea. It has a warm-temperate continental arid climate, with arid and hot summers and cold winters. The average annual rainfall is 100-200 mm,

most of which falls in the summer, making it a highly arid zone. Cultivated and unutilized land account for the largest proportions of land use types in the study area (Standard for Classification of Current Land Use Situations (GBT21010-2007)). The terrain is high in the north and low in the south. Due to the Western development strategy, the population increased from 1.88×10^4 in 1990 to 2.39×10^4 in 2020, and the GDP grew from 4.62×10^8 CNY to 1.85×10^{10} CNY. Therefore, the study area's ecological pattern underwent a significant change. The expansion of cultivated land and the development of unutilized land significantly contributed to regional economic growth. However, rapid development has also put tremendous pressure on fragile ecosystems.

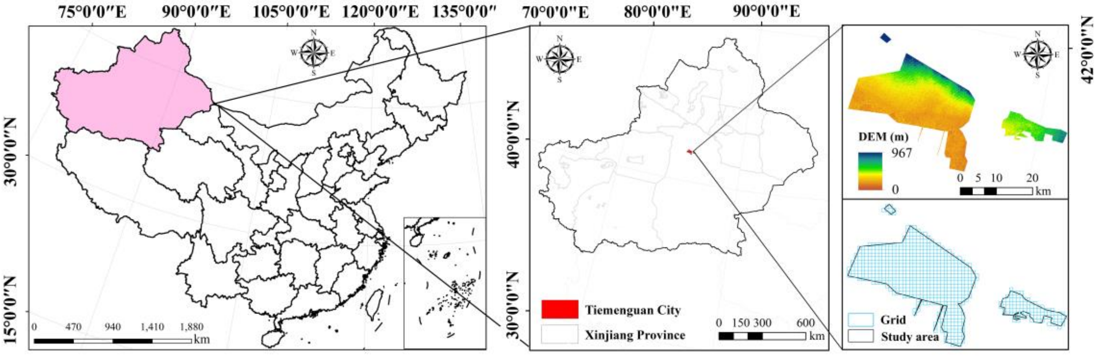

Fig. 2. Location of the study area.

2.2. Data sources

We used three data sources:

(1) Remote sensing images: We used Landsat series images from 1990 to 2020 (with an interval of 10 years). We selected images from June to September with less than 10% cloud cover. The NDVI was used to classify oasis, transition, and desert zones. Misclassifications were corrected using land-use data and high-resolution satellite images (Fig. S1). The steps were as follows: (a) ENVI 5.3 was used to pre-process the images using splicing, cropping, radiometric calibration, and atmospheric correction. The NDVI of the images from 4 dates (resolution of 30 × 30m) was calculated. (b) Google Earth high-resolution images were used to determine the NDVI thresholds between the oasis,

transition, and desert zones (NDVI ≥ 30% for the oasis zone, 10 < NDVI < 30% for the transition zone, and NDVI ≤ 10% for the desert zone). (c) The classification errors were corrected using visual interpretation and land use data. (d) The accuracy of the classification results was determined by selecting 2000 random points and using Google Earth high-resolution images. The OA and Kappa coefficient were above 80%, meeting the research requirements (Table 1).

(2) Land use data: Land use data were obtained from the Center for Resource and Environmental Science and Data of the Chinese Academy of Sciences (www.resdc.cn).

(3) Geographic data: We used GDP, population, elevation (digital elevation model (DEM), slope, soil erosion, average annual temperature, annual rainfall, and distance from roads and towns. The distances were extracted using Euclidean distance analysis in ArcGIS 10.8. Table 2 lists the data sources.

Table 1. Classification accuracy.

Year		Oasis	Transition zone	Desert	UA	PA	OA	Kappa
1990	Oasis	536	66	3	0.91	0.89	0.90	0.84
	Transition zone	34	436	54	0.81	0.83		
	Desert	16	33	822	0.94	0.94		
2000	Oasis	566	41	7	0.88	0.92	0.91	0.86
	Transition zone	49	388	30	0.85	0.83		
	Desert	29	29	861	0.96	0.94		
2010	Oasis	748	21	15	0.93	0.95	0.91	0.86
	Transition zone	43	382	40	0.86	0.82		
	Desert	16	41	694	0.93	0.92		
2020	Oasis	1024	26	23	0.97	0.95	0.92	0.86
	Transition zone	23	273	44	0.82	0.80		

Note: OA: overall accuracy; Kappa: kappa coefficient; UA: user accuracy; PA: producer accuracy.

Table 2. Geographic data.

Data usage	Category	Data index	Data Resource
		DEM	Geospatial Data Cloud
		Slope	(https: //www.gscloud.cn/)
	Natural factor	Temperature	National Centre for Atmospheric Science
		Precipitation	(https: //ncas ac.uk/)
Assessment of ERIs		Soil erosion	
		GDP	Resource and Environmental Science and Data Center (https: //www.resdc.cn)
	Socio-economic factor	Population	
		Distance from road	OpenStreetMap
		Distance from town	(https: //www.openhistoricalmap.org)

2.3. Calculation of ERI

An ecological risk assessment identifies and quantifies potential risk factors [29] . The ERI has been widely used in ecological risk assessments and studies of changes in the landscape structure [30] . We analyzed the spatial distribution and evolution of different landscape types in the region to assess the risk to oasis urban ecosystems. The ERI was established using the LDI, Landscape Fragility Index (LFI), and Landscape Ecological Loss Degree (LLD) to determine the relationship between regional ecological risk and land use type [31] . A 1 km × 1 km grid (Fig. 2) was created in ArcMap 10.8 to delineate the study area. Spatial interpolation was used to determine the risk in each grid. The equations for the indices are as follows:

$$C_i = \frac{N_i}{A_i} \tag{1}$$

$$S_i = \frac{A}{2A_i}\sqrt{\frac{N_i}{A}} \tag{2}$$

$$D_i = \frac{Q_i + M_i}{4} + \frac{L_i}{2} \tag{3}$$

$$U_i = a \times C_i + b \times S_i + c \times D_i \tag{4}$$

$$R_i = U_i \times F_i \quad (5)$$

$$ERI_k = \sum_{i=1}^{n} \frac{A_{ki}}{A_k} R_i \quad (6)$$

where C_i denotes the landscape fragmentation index; N_i and A_i represent the number of patches in landscape i and the area of landscape i, respectively; S_i is the landscape separateness index; A is the total area; D_i is the landscape dominance index; Q_i, M_i and L_i represent the ratio of the number of samples to the total number of samples in landscape i, the ratio of the number of patches to the total number of patches in landscape i, and the ratio of the area of the number of patches to the total area in landscape i, respectively; a, b, and c are the weights of the landscape indicators. Their values were based on previous experience: a = 0.5, b = 0.3, and c = 0.2 (for unutilized land, the weights were 0.3, 0.2, and 0.5, respectively) [32, 33]. R_i, U_i, and F_i denote the LLD, LDI, and LFI, respectively. The LFI was calculated as follows: the six landscape types were assigned vulnerability values from high to low (construction land (6), forest land (5), arable land (4), grassland (3), water (2), and desert (1)). The data were normalized to obtain the vulnerabilities of different landscape types [32]. ERI_k is the ecological risk index of the kth sampling area, A_{ki} is the area of landscape type i in the kth sampling area, and A_k is the total area of the kth sampling area.

This study used a spatial autocorrelation model (via GeoDa software) to analyze the global and local autocorrelation of ecological risk and reveal the spatial distribution characteristics of the ERI [34]. Spatial heterogeneity and similarity were quantified using the global Moran's Index. Values close to 1 indicate strong positive spatial autocorrelations between the ecological risk eigenvalues, values close to -1 indicate a negative correlation and a value of o indicates no significant spatial autocorrelation [35]. Spatial clustering of high and low ERI values based on the global Moran's

Index was used to classify the study area into four types: High-High, High-Low, Low-High, and Low-Low to reveal the spatial aggregation and dispersion patterns of ecological risks. The equations are as follows [33]：

$$GlobalMoran's\ Index = \frac{n\sum_{i=1}^{n}\sum_{j=1}^{n} w_{ij}(x_i-\bar{x})(x_j-\bar{x})}{(\sum_{i=1}^{n}\sum_{j=1}^{n} w_{ij})\sum_{i=1}^{n}(x_i-\bar{x})^2} \quad (7)$$

$$LocalMoran's\ Index = \frac{n(x_i-\bar{x})\sum_{j=1}^{n} w_{ij}(x_j-\bar{x})}{\sum_{i=1}^{n}(x_i-\bar{x})^2} \quad (8)$$

where n is the number of samples, x_i and x_j denote the attribute values of i and j, respectively, $\bar{x}$ is the mean value, and w_{ij} is the spatial weight matrix.

2.4. Driving mechanisms of ecological risk

This study investigated the driving mechanism of ecological risk using an RF regression model (shown in Fig. S2) [33]. RF is an ensemble algorithm with a better learning performance than a single decision tree model. The model determines the importance of the covariates. This method has been widely used in many fields, such as data mining and ecology. The algorithm uses bootstrap sampling, where N training sets are sampled with replacement (about 2/3 of the total number of samples), and the results are evaluated using the remaining samples as a test set. Multiple decision trees are generated using the training set, and m of the M feature variables are randomly selected as node divisions. The prediction results of the N decision trees are used to determine new sample categories by voting. An RF model with N decision trees is constructed and scored by the test set, and the variable importance is calculated. The feature importance score (IS) is calculated as follows：

$$IS = \frac{\sum(errOOB2-errOOB1)}{N} \quad (9)$$

Each decision tree's out-of-bag data *error (errOOB1)* is computed, and *errOOB2* is the error after random noise interference is added to all sample features of the out-of-bag data.

The model parameters were screened using a grid search and 4-fold cross-validation to improve

model performance [36]. The parameter ranges were as follows: n_estimators ranged from 5-50 in steps of 1, max_depth ranged from 1-20 in steps of 1, max_features ranged from 1-10 in steps of 1, and the random_state was 1. The process was implemented in MATLAB R2023a.

The parameters used for model evaluation were the determination coefficient (R^2), root mean square error (RMSE), and relative percent deviation (RPD). When RPD ≥ 2, The model performance is excellent when RPD ≥ 2, fair when 1.4 ≤ RPD < 2, and low when RPD < 1.4[37].

$$R^2 = \frac{\sum_{i=1}^{n}(\hat{y_i}-\bar{y_i})^2}{\sum_{i=1}^{n}(y_i-\bar{y_i})^2} \quad (10)$$

$$RMSE = \sqrt{\frac{\sum_{i=1}^{n}(\hat{y_i}-y_i)^2}{n}} \quad (11)$$

$$RPD = \sqrt{\frac{\sum_{i=1}^{n}(y_i-\bar{y})^2}{\sum_{i=1}^{n}(\hat{y_i}-y_i)^2}} \quad (12)$$

where y is the measured value of ERI, $\hat{y_i}$ is the predicted value of ERI, $\bar{y_i}$ is the mean value of ERI content, and n is the total number of samples (i=1, 2, 3...n).

We conducted a quantitative analysis of the contribution of ecological risk as a driving mechanism. The ERI values in 2000, 2010, and 2020 (1990 was not analyzed due to incomplete data) were used as the dependent variable. Elevation, Slope, Soil erosion, Temperature, Precipitation, GDP, Population, Distance from roads, and Distance from towns were used as independent variables. The 646 sample points were randomly divided into a training set (484) and a validation set (162). The original data were linearly normalized to ensure model stability.

2.5. Predicting changes in land use types, OLS, and ERI

We used the PLUS model (version 1.3.5) to simulate future land use change scenarios. The model combines meta-CA and patch generation simulation and contains modules for land expansion extraction, land expansion analysis strategy (LEAS), demand forecasting, and scenario diversity. The LEAS extracts the land use changes in two periods, analyzes the driving factors using the RF

algorithm, and obtains the development probability of each type. The number of regression trees was 40, and the sampling rate and number of RF features were 0.01 and 12, respectively. The PLUS model dynamically simulates the generation of patches under the constraint of development probability[38, 39].

Before the simulation, the Markov chain was used to predict land changes, and the results were used as model parameters. The land use changes in the study area in 2020 were simulated based on the land use data in 2000 and 2010. We compared the results with actual conditions in 2020 and obtained a Kappa coefficient of 0.90 and an OA of 0.94, indicating the model's reliability for subsequent analyses.

Considering the realities of arid oases and the relevant literature， we simulated the land use change patterns under a natural growth scenario and a government regulation scenario and analyzed the spatial distribution characteristics of ERI and the quantitative characteristics of OLS under different scenarios [14, 33]. In the natural development scenario, all land use demands are simulated based on the Markov chain model (no change). By the Markov chain model, the government regulation scenario, the transferring of cropland, woodland, and grassland to built-up land, and unused land decreased by 20%, the transferring unused land to cropland, woodland, and grassland increased by 30%, and the transferring built-up land to woodland and grassland increased by 10%.

3. Results

3.1. Spatial and temporal evolution of OLS and ERI, 1990-2020

3.1.1. Spatial and temporal evolution of OLS

From 1990 to 2020, the oasis area expanded from the core area to the outside, significantly reducing desert areas in all directions. Over time, many oasis patches expanded, connected, and

formed a large contiguous oasis belt. The trend exhibited stages with small and large rates of change (Fig. 3a, Table 3). The period from 1990 to 2000 (the first stage) showed a small rate of change in the oasis zone (about 8.5% growth) and a trend of expansion in the desert zone (about 4.3% expansion), with limited spatial expansion of the elements. The change rate in the landscape structure increased significantly, with the oasis zone expanding from 190.6 km^2 to 345.3 km^2 and the transition and desert zones declining significantly by 38.7% and 40.3%, respectively.

The differences in spatial transitions between the oasis, transition, and desert zones were evident from 1990 to 2020 (Fig. 3b). In the first stage, the conversion from desert to oasis was not significant (changes in all factors were below 38 km^2), indicating that the effectiveness of ecological management was limited during this period, and the land degradation trend was not significantly curbed. Conversely, the second phase was a critical period of oasis expansion, with oases acquiring a significant area from transition zones and deserts (81.67 and 36.4 km^2, respectively). The desert area decreased significantly from 2010 to 2020 and was transformed into transition and oases zones, with areas of 41.02 and 31.38 km^2, respectively.

The region's ecological environment improved significantly from 1990 to 2020, and especially after 2000. The oasis area expanded, and deserts were transformed into oasis and transition zones. This trend shows that land degradation was controlled by effective ecological management measures during the past 30 years.

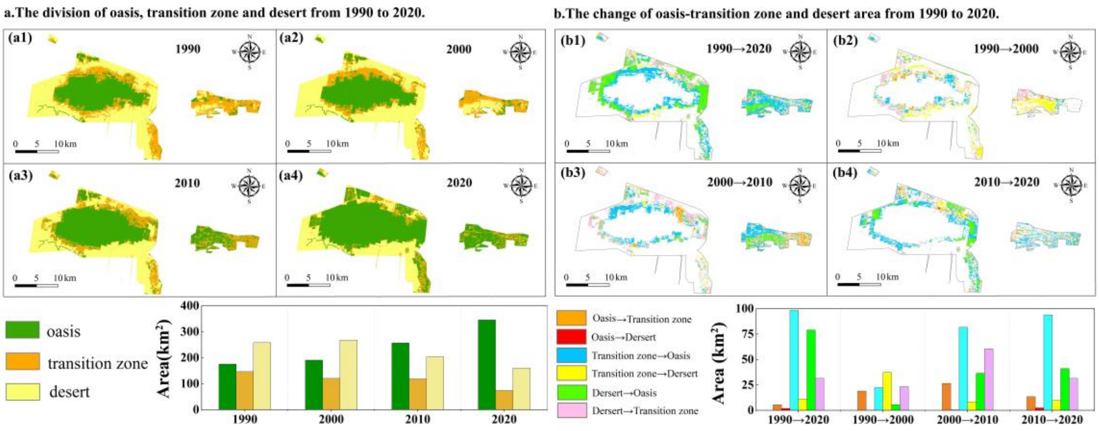

Fig. 3. Areas of oasis, transition, and desert zones and shifts in landscape patterns from 1990 to 2020.

Table 3. Area statistics for of oases, transition, and desert zones and changes in landscape patterns from 1990 to 2020.

Year	Oasis	Transition zone	Desert	Oasis→Transition zone	Oasis→Desert	Transition zone→Oasis	Transition zone→Desert	Desert→Oasis	Desert→Transition zone
1990	175.5	146.3	257.8	/	/	/	/	/	/
2000	190.6	120.4	268.6	/	/	/	/	/	/
2010	256.8	118.9	204.0	/	/	/	/	/	/
2020	345.3	73.6	160.0	/	/	/	/	/	/
1990-2020	/	/	/	5.54	1.93	98.25	11.14	78.94	31.45
1990-2000	/	/	/	18.96	0.21	23.22	37.27	5.44	23.35
2000-2010	/	/	/	26.51	0.42	81.66	8.43	36.39	60.44
2010-2020	/	/	/	13.49	2.42	93.74	9.93	41.02	31.38

3.1.2. Spatial and temporal evolution of land-use type and transfer

A comprehensive analysis of 30 years of land use data and its changes (Table 4) shows that the study area has a high percentage of cropland, medium to high-cover grassland, and unused land (more than 90% of the total). Due to the implementation of the Western Development Strategy, the land use change from 2000 to 2020 differed significantly from that of the previous decade (Fig. 4a).

From 1990 to 2020, cropland and construction land increased by 40.84% and 342.29%, and forest land and grassland decreased by 37.60% and 53.96%, respectively. More land use change occurred in the southern and eastern regions of the study area, where the production capacity of arable land declined, and land degradation was not effectively curbed. Figure 4b depicts the magnitude and direction of land-use change over time, indicating substantial cross-regional shifts between unutilized land and grassland.

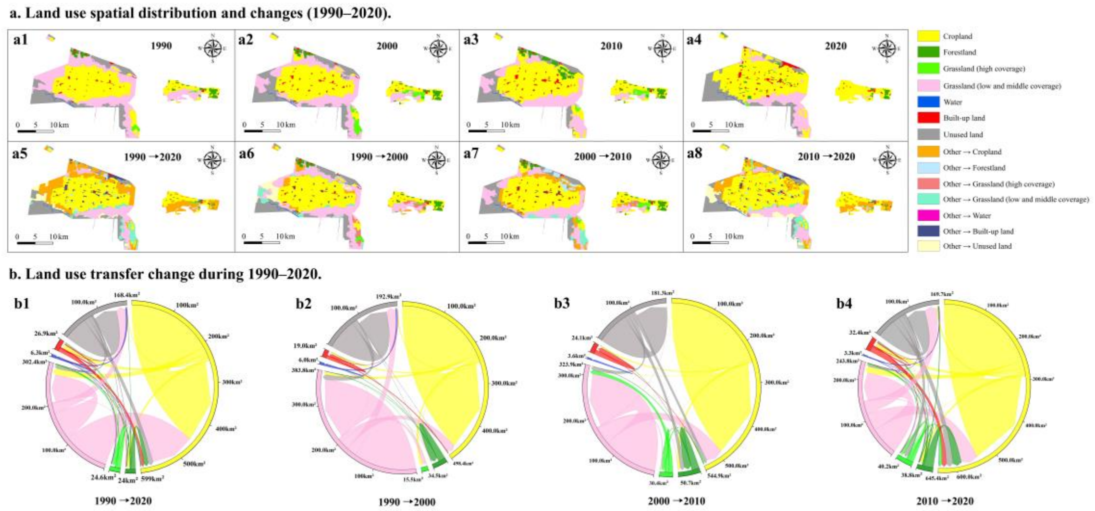

Fig. 4. Land use change from 1990 to 2020.

Table 4. Areas of different land use types from 1990 to 2020 (km^2).

Year	Cropland	Forestland	Grassland (high coverage)	Grassland (low and middle coverage)	Water	Built-up land	Unused land
1990	247.48	16.94	4.43	209.33	2.57	8.37	88.75
2000	250.94	17.58	19.89	174.49	1.80	13.66	104.10
2010	294.00	33.13	10.46	149.41	1.80	23.47	77.19
2020	348.56	10.57	1.01	86.37	1.79	37.02	94.99

3.1.3. Spatial and temporal evolution of ERI

The spatial distribution and trend of the ERI and Moran's Index from 1990 to 2020 (Fig. 5) indicate a significant reduction in high ecological risk areas, especially in the eastern part of the

study area, suggesting that rational land planning has improved ecological conditions. Low ERI areas increased over time, indicating a broad reduction in ecological risk and a transition from high-risk to low-risk areas in multiple locations. This improvement likely resulted from targeted interventions, such as land management reforms. The ERI levels are significantly lower in the central part of the study area, indicating that this region is ecologically stable and has low risk. Ecological risk management in this region has shown favorable results after 30 years. The percentage change of areas with different ERI levels is shown in Fig. 5a. The percentage of high-risk areas was 21.41% in 1990 and decreased significantly (6.8%) after 30 years. The percentage of low-risk areas increased significantly, indicating that the region's ecological risk was mitigated. The ERI exhibited variability during the 30-year period (Fig. 5b). From 1990 to 2000 (Fig. 5b-I), regions with High-High ERI values were concentrated at the periphery of the study area, whereas Low-Low regions were primarily located in the center of the oasis. In contrast, the High-High areas decreased significantly in the 2010-2020 period (Fig.5b-K), whereas the Low-Low areas expanded significantly, indicating a reduction in ecological risk. Figure 5c shows the spatial distribution of the global and local Moran's indices. Spatial clustering of ecological risks increased during the 30-year period (the global Moran's Index increased from 0.717 to 0.792). Regions with Low-Low values in the eastern part of the study area increased sharply from 2000 to 2020, and the degree of ecological fragmentation decreased significantly. The distribution of high-risk and low-risk areas exhibited negligible change over time, and the spatial heterogeneity of risk increased. These results suggest that land-use changes and ecological management measures have mitigated ecological risks in the region. However, local clustering of high-risk areas was observed.

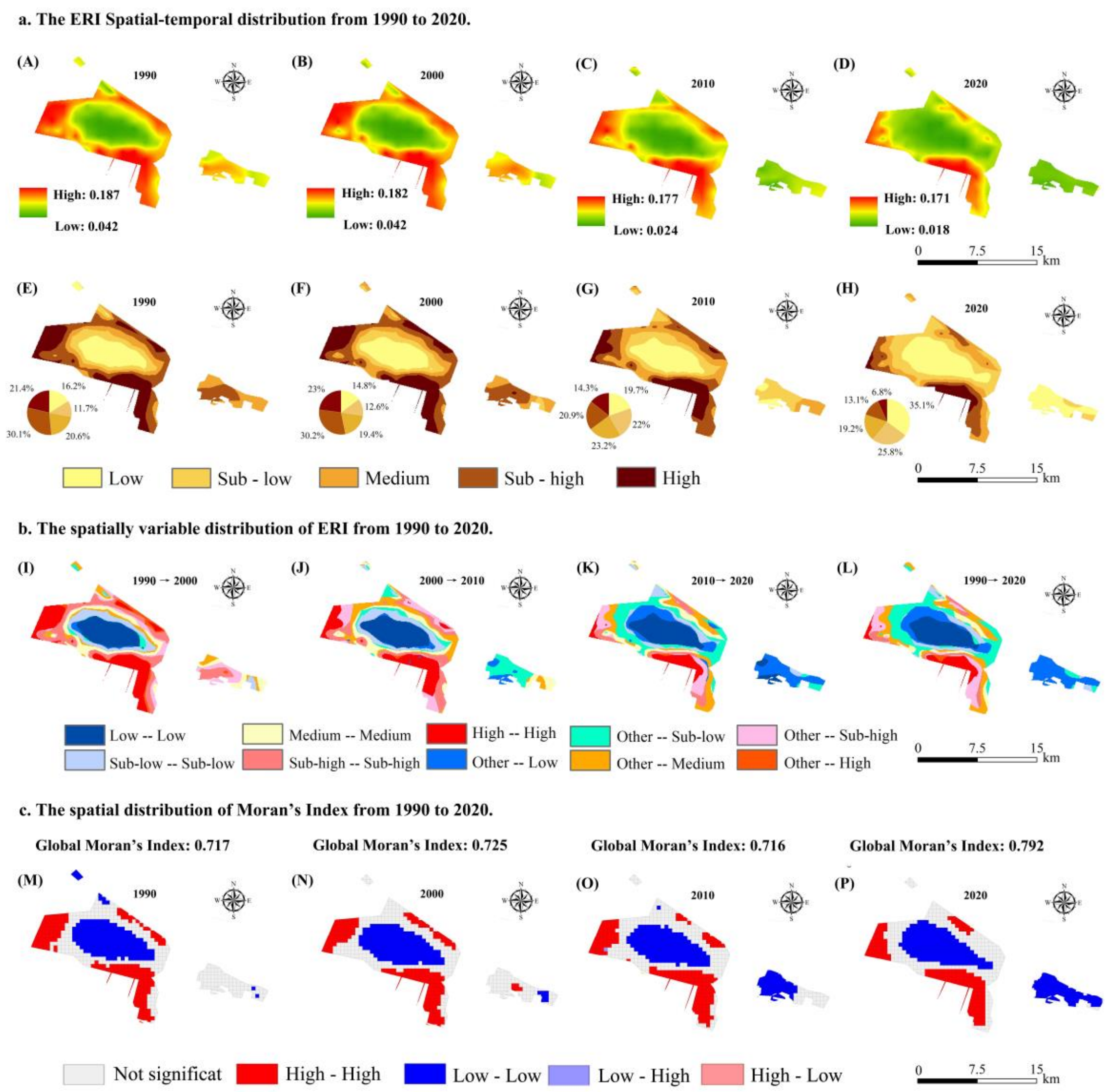

Fig. 5. Spatiotemporal distribution of ERI in oasis cities (1990—2020).

3.1.4. Potential link between ERI and OLS

The ERI characteristics of the oasis, transition, and desert zones varied significantly in different periods (Fig. 6). The ERIs differed significantly in different OLS regions, suggesting a potential link. The ERI was significantly lower in the oasis zone (the mean value was 0.05 to 0.06), suggesting a stable ecosystem. The ERI of the transition zone was intermediate (the mean value was 0.085 to 0.102) and fluctuated significantly, demonstrating that the ecological risk was high and the region was vulnerable to environmental influences and land use changes. The ERI value of the desert zone

was high (the average value was 0.127 to 0.144), and the change was relatively small, reflecting the high vulnerability of desert regions. The ERI in the transition and desert zones exhibited a fluctuating downward trend throughout the period. It was the lowest in 2020, indicating that environmental protection measures had been successful.

We selected 1000 random sample points at the borders of the oasis zone-transition zone and the transition zone-desert zone to calculate the ERI and OLS thresholds in adjacent landscape structures. The standard deviation was used to exclude outliers. The results are listed in Table 6. The thresholds of the oasis zone-transition zone are in the range of 0.08-0.085 and decrease over time, and the thresholds of the transition zone-desert zone show the same trend (the ERI has a range of 0.111-0.118). Both thresholds exhibit slight fluctuations and are affected by the OLS characteristics.

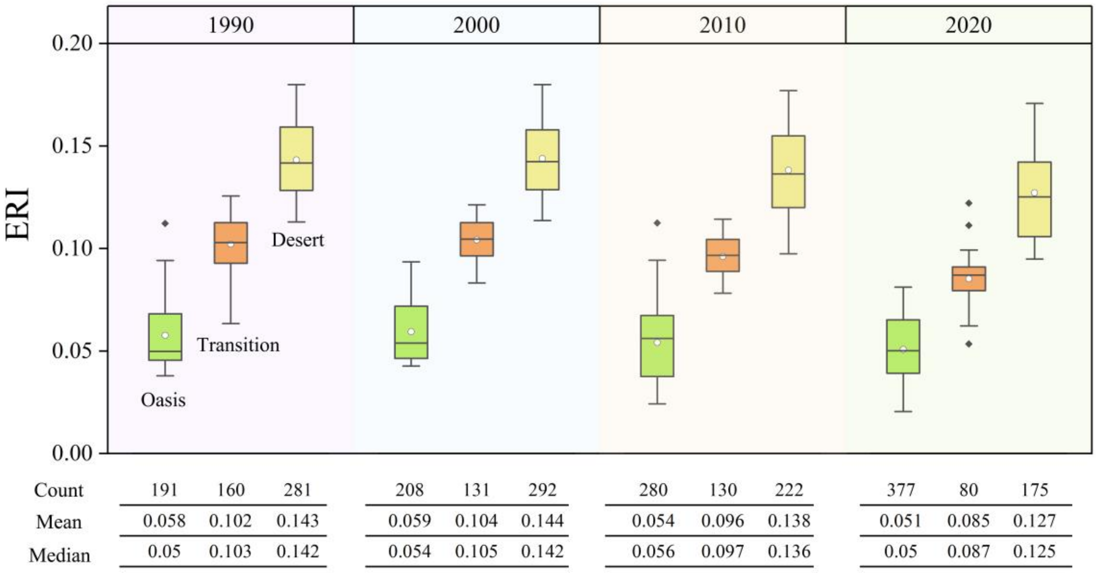

Fig. 6. Quantitative characteristics of ERI in different OLSs from 1990 to 2020.

Table 6. Threshold of ERIs corresponding to changes in each category in OLS.

Year	1990	2000	2010	2020
Oasis—Transition zone	0.084	0.085	0.082	0.08
Transition zone—Desert	0.116	0.118	0.114	0.111

3.2. Spatiotemporal factors affecting the ERI

The observed and predicted R^2 values of the validation set of ERI were 0.73, 0.71, and 0.77 in 2000, 2010, and 2020, respectively, indicating that the RF regression model has high predictive ability. The RMSE values ranged from 0.0136 to 0.0165, and the RPD values ranged from 1.79 and 2.15, demonstrating the model's high stability (Fig. 7a). The distances to towns and roads had the most significant influence on the ERI in 2000 (relative influences of 0.21 and 0.17, respectively), whereas the slope and elevation had the smallest influences (Fig. 7b). The socioeconomic factors remained dominant from 2000 to 2010, but the relative importance of natural factors, such as precipitation and temperature, declined over time. The results for 2020 confirm the dominance of socioeconomic factors and the continued decline in the influence of natural factors on the ERI.

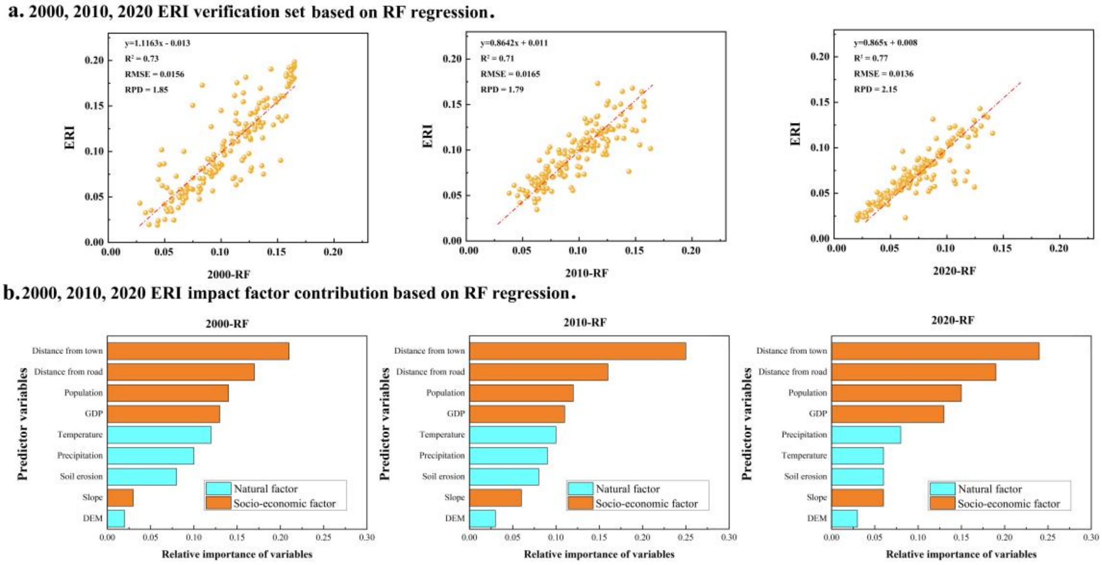

Fig. 7. Results of RF regression model validation and significance of influencing factors (desert area change and spatiotemporal characteristics of ERI).

3.3. Projections of trends in ERI and OLS

3.3.1 Projections of trends in land-use change

The actual and projected land use is shown in Fig. 8a. The difference between the actual and

simulated land use types in 2020 was low than 3.5%. Cropland accounted for 59.3% of the total area of the simulated land use types, and unutilized land and grassland with medium to low cover accounted for 32.3%. The simulated and actual data had similar proportions of grassland, built-up land, and unutilized land. The area of cultivated land increased by 62.3% and 59.7% in the natural growth and government control scenarios, respectively, from 2020 to 2030, whereas the areas of low-cover and medium-cover grassland decreased. Forest land and high-cover grassland showed an upward trend, and the proportion of forest land and grassland increased in the government control scenario. The prediction results show that effective government management has limited the expansion of cultivated land and improved ecological stability.

3.3.2 Prediction of trends in the ERI

The projected spatial pattern of the ERI in 2030 is shown in Fig. 8b. Under the natural growth scenario, the low-risk area of ERI is concentrated in the center of the study area, accounting for 36.3% of the area. The high-risk areas account for 4.7% of the area and are primarily located in the southern part of the study area. In contrast, the area proportion of low-risk areas increases to 39.6%, and the area share of high-risk areas decreases to 4.5% under the government control scenario. In this scenario, more low-risk areas occur, and the risk of the oasis ecosystem is significantly reduced.

3.3.3 Prediction of trends in the OLS

The proportion of land use types in oasis, transition, and desert zones in 2020 is listed in Table 7. Based on the trend of the simulated OLS components in 2030 (Fig. 8c), the oasis area increased from 2020 to 2030 under natural and controlled scenarios, reaching 382.6 km^2 and 375.3 km^2, respectively. The transition zone area increased significantly under the government-controlled scenario, reaching 82.5 km^2, much higher than that under the natural growth scenario, indicating

that government interventions improved the ecological restoration of oasis margins. The desert area decreased under both scenarios from 2020 to 2030, indicating the importance of conservation for biodiversity and limiting land expansion.

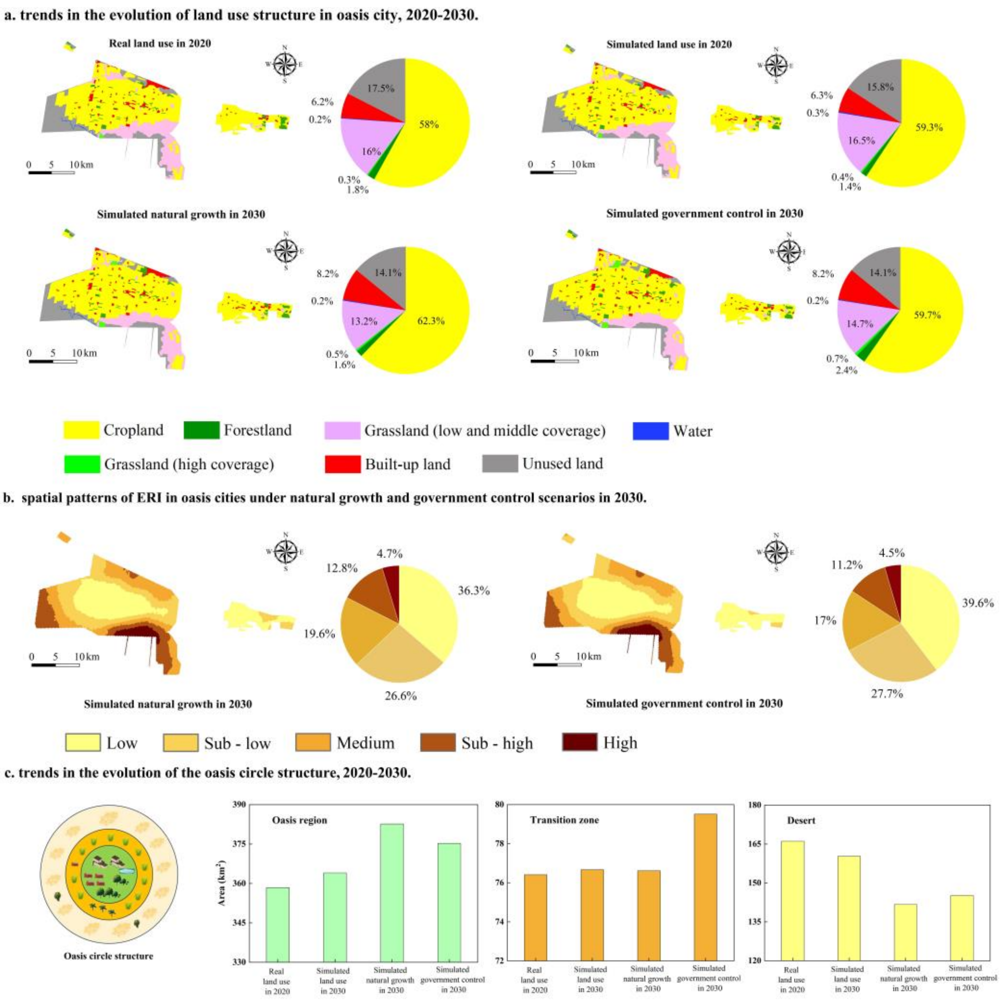

Fig. 8. Projected land use, ERI and OLS in 2030 under different conditions.

Table 7. Proportion of land use types in oasis, transition, and desert zones in 2020.

	**Category**	Oasis	Transition zone	Desert
	Cropland	88.0	12.0	0.0
	Forestland	85.8	13.2	0.9
	Grassland (high coverage)	59.4	9.9	29.7
Percentage/%	Grassland (low and middle)	11.8	20.4	67.7
	Water	100.0	0.0	0.0
	Built-up land	100.0	0.0	0.0
	Unused land	0.0	10.1	89.9

4. Discussion

4.1 Potential link between OLS and ERI

The ERIs of different OLS regions were significantly different, suggesting a correlation between ERI and OLS (Fig. 6). The average ERIs of oasis, transition, and desert zones were 0.05-0.06, 0.085-0.102, and 0.127-0.144 in 1990 to 2020, respectively, indicating an increasing trend. This result is likely due to differences in regional ecological management, the impacts of environmental disturbances, and differences in ecosystem resilience[40, 41]. Oasis zones have lower ecological risks due to higher vegetation cover and more scientific management. In contrast, transition and desert zones are more vulnerable to the negative impacts of climate change and human activities due to sparse vegetation and inadequate management [42, 43]. In addition, the oasis area has a more robust ecosystem and is more resilient than the desert and transition zones. The concentration of human activities and inputs has reduced ecological risks in the oasis area. These factors contribute to the significant differences in ecological risk levels in different regions [44]. In addition, OLS and ERI show similar spatial patterns, i.e., a circular structure, reflecting the spatial characteristics of the oasis zone (low ERI), transition zone (medium ERI), and desert zone (high

ERI) (Fig. 8c). These results are related to the gradient, the concentration of human activities, and differences in ecosystem resilience [44, 45].

4.2 Drivers of the ERI spatial pattern

The spatiotemporal evolution of the ERI exhibits significant heterogeneity, which, combined with the results of the empirical analysis, is influenced by natural and socioeconomic factors (Fig. 7). We analyzed the dynamic evolution of regional ecological risk affected by natural and socioeconomic factors at different temporal scales. First, the influence of topographic relief (e.g., slope and elevation) was more stable (the combined relative influence was below 0.1). This factor affects soil erosion, contributing to the distribution of the ERI [33, 46]. Second, the effects of socioeconomic factors, such as infrastructure expansion, population density, and GDP growth, on the ERI became more dominant over time [47] . Human activities alter ecosystem stability through land use change and environmental modifications. Thus, the ERI decreased from 0.041-0.182 (2000) to 0.018-0.171 (2020) (Fig. 5). The expansion of roads and towns during urbanization significantly reduced the ERI [48]. The relative importance of climatic factors, such as precipitation and temperature, declined over the past decades, which may be related to improved infrastructure and regional adaptive management measures [49].

4.3 Impacts of land use change on OLS and ecological risk

Land use change directly affects the evolution of the OLS [50, 51]. The expansion of cultivated and built-up land and the reduction of unutilized land increased the oasis zone from 175.5 km^2 in 1990 to 190.6 km^2 in 2000, increasing ecological risk. Human activities intensified during this period due to economic development, and forest land was cleared for agriculture and construction. The lack of effective ecological management increased the ecological vulnerability of

the transition and desert zones [52]. Insufficient vegetation cover weakened the transition zone's buffering ability, reducing ecosystem stability in some areas and landscape connectivity [53]. Optimizing land-use management improved the OLA significantly and reduced ecological risk from 2000 to 2020. Due to ecological protection policies, more unutilized land was converted into oasis areas, and vegetation restoration and land management improved ecosystems, especially in transition zones and desert areas [54]. This change resulted in an expansion of the oasis (190.6 km^2 to 345.3 km^2), an increase in landscape connectivity, and a downward trend in the ERI from 0.182 to 0.171. Effective land use management slowed desertification and improved ecosystem resilience.

4.4 Limitations and future research

This study simulated land use change using the PLUS model. Although this model has high predictive accuracy, it is limited by existing conditions. In addition, it may not fully capture unexpected changes in socioeconomic or environmental conditions [38, 55].

We chose only one oasis desert city in Xinjiang, Tiemenguan City, for our study. However, this region's unique geographic and climatic characteristics may limit the conclusions' applicability to other arid and semi-arid regions. Tiemenguan City has the advantage of being a typical oasis desert city that underwent significant ecological changes. Thus, the results provide insights into dynamic desertification and oasis expansion. The region is little affected by the outside world, and the influence of external variables on the research results is minor. Therefore, Tiemenguan City is a representative case that reveals the changes and driving mechanisms of the oasis desert urban landscape structure.

More in-depth research on the landscape structure of oasis desert areas can be conducted by utilizing our research results. Various socioeconomic and climatic scenarios can be assessed to

understand the risks and challenges of ecosystems in oasis desert areas under different development paths [56, 57]. We plan to expand the research scope to other oasis desert zones and conduct a cross-regional comparative study to reveal the ecological risk-driven mechanism under different geographic and socioeconomic backgrounds and provide references for the ecological management of arid and semi-arid oasis desert zones.

5. Conclusion

This study examined the spatiotemporal evolution characteristics of the OLS and ERI from 1990 to 2020 and their potential correlation. We used RF regression models to analyze the drivers of spatial changes in the ERI and the PLUS model to predict the trends of land-use change and the spatial characteristics of ERI and OLS in 2030 under natural development and government control scenarios. The following results were obtained. (1) The oasis zone expanded from 175.5 km^2 to 345.3 km^2, and the desert zone decreased by 37.9% from 1990 to 2020. The ERI decreased, and the thresholds for changes in the ERI of the oasis-transition zone and the transition zone-desert were 0.08-0.085 and 0.111-0.118, respectively. (2) Socioeconomic factors were the dominant factors affecting the ERI, and the trend was constant. The influence of natural factors on the ERI decreased in each period. The relative influence of the distances to towns and cities was the largest (exceeding 0.21), while the relative influence of elevation was the smallest. (3) More unutilized land was transformed into cropland by 2030 (from 58% to 62.3%) under the natural scenario. The ecological risk level decreased (the proportion of High-ERI areas will decrease by 2.1%), the oasis area increased (to 382.6 km^2), and the transition and desert areas decreased. The expansion rate of cropland decreased under the government-controlled scenario (reaching 59.7%). The ecological risk was lower in the government-controlled scenario than in the natural scenario, and the growth rate

of the oasis area slowed. The proportion of the transition zones increased significantly. In summary, in optimizing natural resources and choosing suitable boundaries are recommended in oasis desert areas with relatively harsh environments to achieve a regional balance between human needs and ecosystem conservation.

Acknowledgments

We thank the School of Water Resources and Architectural Engineering, Shihezi University, and the Hydrology and Water Resources Management Center of the Second Division of the Xinjiang Production and Construction Corps.

Supporting information

S1 File. Flow chart of extraction oasis-desert transition zone-desert. Note: LUD denotes land use data.

S2 Fig. Flowchart of a Random Forest structure with cross-validation.

References

1. Liu L, Xue J, Mao DL, Chang JJ, Wang SK, Li XX. An integrative socio-hydrological resilience assessment and management implications for oasis sustainability in arid regions, Northwest China. J HYDROL-REG STUD. 2023;47. doi: 10.1016/j.ejrh.2023.101389. PubMed PMID: WOS:001044634600001.
2. Xiao FY, Gao GY, Shen Q, Wang XF, Ma Y, Lü YH, et al. Spatio-temporal characteristics and driving forces of landscape structure changes in the middle reach of the Heihe River Basin from 1990 to 2015. Landscape Ecology. 2019;34(4):755-70. doi: 10.1007/s10980-019-00801-2. PubMed PMID: WOS:000467558400004.
3. Zhao YY, Kasimu A, Gao PW, Liang HW. Spatiotemporal Changes in The Urban Landscape Pattern and Driving Forces of LUCC Characteristics in The Urban Agglomeration on The Northern Slope of The Tianshan Mountains from 1995 to 2018. Land. 2022;11(10). doi: 10.3390/land11101745. PubMed PMID: WOS:000872832800001.
4. Li BF, Gui DW, Xue DP, Liu YF, Ahmed Z, Lei JQ. Analysis of the Expansion Characteristics and Carrying Capacity of Oasis Farmland in Northwestern China in Recent 40 Years. Agronomy-Basel. 2022;12(10). doi: 10.3390/agronomy12102448. PubMed PMID: WOS:000875895600001.
5. Wang JG, Zhang F, Jim CY, Chan NW, Johnson VC, Liu CJ, et al. Spatio-temporal variations and drivers of ecological carrying capacity in a typical mountain-oasis-desert area, Xinjiang, China. ECOL ENG. 2022;180. doi: 10.1016/j.ecoleng.2022.106672. PubMed PMID: WOS:000802081100003.
6. Ding H, Xingming H. Spatiotemporal change and drivers analysis of desertification in the arid region of northwest China based on geographic detector. Environmental Challenges. 2021;4:100082. doi: https://doi.org/10.1016/j.envc.2021.100082.
7. Zhang QF, Sun CJ, Chen YN, Chen W, Xiang YY, Li J, et al. Recent Oasis Dynamics and Ecological Security in the Tarim River Basin, Central Asia. Sustainability. 2022;14(6). doi: 10.3390/su14063372. PubMed PMID: WOS:000774425500001.
8. Zhang W, Ma L, Li H, Wang XJL, Planning U. Trends in the future evolution of rural settlements in oasis-desert areas under water use simulation scenarios: Take the Hexi Corridor region of China as an example. 2024;248:105110.
9. Gao H, Song W. Assessing the Landscape Ecological Risks of Land-Use Change. INT J ENV RES PUB HE. 2022;19(21). doi: 10.3390/ijerph192113945. PubMed PMID: WOS:000881195700001.
10. Wang Y, Yang Z, Yu M, Lin R, Zhu L, Bai F. Integrating Ecosystem Health and Services for Assessing Ecological Risk and its Response to Typical Land-Use Patterns in the Eco-fragile Region, North China. ENVIRON MANAGE. 2023;71(4):867-84. doi: 10.1007/s00267-022-01742-4. PubMed PMID: WOS:000877394000001.
11. Yang Y, Chen J, Lan Y, Zhou G, You H, Han X, et al. Landscape Pattern and Ecological Risk Assessment in Guangxi Based on Land Use Change. INT J ENV RES PUB HE. 2022;19(3). doi: 10.3390/ijerph19031595. PubMed PMID: WOS:000756392900001.
12. Huang SM, Hu QW, Wang SH, Li HD. Ecological Risk Assessment of World Heritage Sites Using RS and GIS: A Case Study of Huangshan Mountain, China. CHINESE GEOGR SCI. 2022;32(5):808-23. doi: 10.1007/s11769-022-1302-4. PubMed PMID: WOS:000850588200005.
13. Wang DC, Chai H, Wang ZH, Wang KJ, Wang HY, Long H, et al. Dynamic Monitoring and Ecological Risk Analysis of Lake Inundation Areas in Tibetan Plateau. Sustainability. 2022;14(20). doi: 10.3390/su142013332. PubMed PMID: WOS:000873724000001.

14. Song Q, Hu B, Peng J, Bourennane H, Biswas A, Opitz T, et al. Spatio-temporal variation and dynamic scenario simulation of ecological risk in a typical artificial oasis in northwestern China. J CLEAN PROD. 2022;369. doi: 10.1016/j.jclepro.2022.133302. PubMed PMID: WOS:000844996300001.
15. Zhao YY, Kasimu A, Liang HW, Reheman R. Construction and Restoration of Landscape Ecological Network in Urumqi City Based on Landscape Ecological Risk Assessment. Sustainability. 2022;14(13). doi: 10.3390/su14138154. PubMed PMID: WOS:000824233100001.
16. Zhang DM, Zhu LB, Ma XY, Liu ZM, Cui HW. Uncoordinated Coupling Assessment of New Urbanization and Ecological Carrying Capacity in the Yellow River Basin. INT J ENV RES PUB HE. 2022;19(15). doi: 10.3390/ijerph19159016. PubMed PMID: WOS:000838956700001.
17. Zhang XY, Liu K, Wang SD, Wu TX, Li XK, Wang JN, et al. Spatiotemporal evolution of ecological vulnerability in the Yellow River Basin under ecological restoration initiatives. ECOL INDIC. 2022;135. doi: 10.1016/j.ecolind.2022.108586. PubMed PMID: WOS:000761305000001.
18. Shen SG, Pu J, Xu C, Wang YH, Luo W, Wen B. Effects of Human Disturbance on Riparian Wetland Landscape Pattern in a Coastal Region. Remote Sensing. 2022;14(20). doi: 10.3390/rs14205160. PubMed PMID: WOS:000873711500001.
19. Xie HL, Zhu ZH, He YF. Regulation simulation of land-use ecological security, based on a CA model and GIS: A case-study in Xingguo County, China. LAND DEGRAD DEV. 2022;33(10):1564-78. doi: 10.1002/ldr.4197. PubMed PMID: WOS:000787486300001.
20. Wang Q, Jiang DY, Gao YF, Zhang ZJ, Chang QR. Examining the Driving Factors of SOM Using a Multi-Scale GWR Model Augmented by Geo-Detector and GWPCA Analysis. Agronomy-Basel. 2022;12(7). doi: 10.3390/agronomy12071697. PubMed PMID: WOS:000831535300001.
21. Xu XH, Yu J, Wang FE. Analysis of ecosystem service drivers based on interpretive machine learning: a case study of Zhejiang Province, China. ENVIRON SCI POLLUT R. 2022;29(42):64060-76. doi: 10.1007/s11356-022-20311-0. PubMed PMID: WOS:000787144700014.
22. Guo HJ, Cai YP, Li BW, Tang YJ, Qi ZX, Huang YP, et al. An integrated modeling approach for ecological risks assessment under multiple scenarios in Guangzhou, China. ECOL INDIC. 2022;142. doi: 10.1016/j.ecolind.2022.109270. PubMed PMID: WOS:000868333000003.
23. Chen X, He XY, Wang SY. Simulated Validation and Prediction of Land Use under Multiple Scenarios in Daxing District, Beijing, China, Based on GeoSOS-FLUS Model. Sustainability. 2022;14(18). doi: 10.3390/su141811428. PubMed PMID: WOS:000856757500001.
24. Gao LA, Tao F, Liu RR, Wang ZL, Leng HJ, Zhou T. Multi-scenario simulation and ecological risk analysis of land use based on the PLUS model: A case study of Nanjing. SUSTAIN CITIES SOC. 2022;85. doi: 10.1016/j.scs.2022.104055. PubMed PMID: WOS:000838106700003.
25. Nasiakou S, Vrahnakis M, Chouvardas D, Mamanis G, Kleftoyanni V. Land Use Changes for Investments in Silvoarable Agriculture Projected by the CLUE-S Spatio-Temporal Model. Land. 2022;11(5). doi: 10.3390/land11050598. PubMed PMID: WOS:000804872700001.
26. Yi SQ, Zhou Y, Li Q. A New Perspective for Urban Development Boundary Delineation Based on the MCR Model and CA-Markov Model. Land. 2022;11(3). doi: 10.3390/land11030401. PubMed PMID: WOS:000775219400001.
27. Yan X, Cheng P, Zhang Q, Li X, He J, Yan X, et al. Comparisons of climate change characteristics in typical arid regions of the Northern Hemisphere. FRONT ENV SCI-SWITZ. 2022;10. doi: 10.3389/fenvs.2022.1033326. PubMed PMID: WOS:000885179400001.
28. Jiang C, Parteli EJR, Xia Q, Yin X, Shao Y. A regional hydrological model for arid and semi-arid

river basins with consideration of irrigation. ENVIRON MODELL SOFTW. 2022;157. doi: 10.1016/j.envsoft.2022.105531. PubMed PMID: WOS:000862467000002.

29. Yang YP, Chen JJ, Lan YP, Zhou GQ, You HT, Han XW, et al. Landscape Pattern and Ecological Risk Assessment in Guangxi Based on Land Use Change. INT J ENV RES PUB HE. 2022;19(3). doi: 10.3390/ijerph19031595. PubMed PMID: WOS:000756392900001.

30. Abbas Z, Zhu ZY, Zhao YL. Spatiotemporal analysis of landscape pattern and structure in the Greater Bay Area, China. EARTH SCI INFORM. 2022;15(3):1977-92. doi: 10.1007/s12145-022-00782-y. PubMed PMID: WOS:000770187600001.

31. Qian Y, Dong Z, Yan Y, Tang LA. Ecological risk assessment models for simulating impacts of land use and landscape pattern on ecosystem services. SCI TOTAL ENVIRON. 2022;833. doi: 10.1016/j.scitotenv.2022.155218. PubMed PMID: WOS:000808120000014.

32. Xue L, Zhu B, Wu Y, Wei G, Liao S, Yang C, et al. Dynamic projection of ecological risk in the Manas River basin based on terrain gradients. SCI TOTAL ENVIRON. 2019;653:283-93. doi: https://doi.org/10.1016/j.scitotenv.2018.10.382.

33. Gan L, Halik Ü, Shi L, Welp M. Multi-scenario dynamic prediction of ecological risk assessment in an arid area of northwest China. ECOL INDIC. 2023;154:110727. doi: https://doi.org/10.1016/j.ecolind.2023.110727.

34. Xu M, Matsushima H. Multi-dimensional landscape ecological risk assessment and its drivers in coastal areas. SCI TOTAL ENVIRON. 2024;908:168183. doi: https://doi.org/10.1016/j.scitotenv.2023.168183.

35. Ai J, Yu K, Zeng Z, Yang L, Liu Y, Liu J. Assessing the dynamic landscape ecological risk and its driving forces in an island city based on optimal spatial scales: Haitan Island, China. ECOL INDIC. 2022;137:108771. doi: https://doi.org/10.1016/j.ecolind.2022.108771.

36. Zhang XW, Chen ZG, Jiao F. Application of the combination method based on RF and LE in near infrared spectral modeling. Spectrochimica Acta Part a-Molecular and Biomolecular Spectroscopy. 2023;289. doi: 10.1016/j.saa.2022.122247. PubMed PMID: WOS:001022607800001.

37. Chen HY, Ma Y, Zhu AX, Wang ZR, Zhao GX, Wei YA. Soil salinity inversion based on differentiated fusion of satellite image and ground spectra. International Journal of Applied Earth Observation and Geoinformation. 2021;101. doi: 10.1016/j.jag.2021.102360. PubMed PMID: WOS:000659161300003.

38. Chen YH, Wang J, Xiong NN, Sun L, Xu JQ. Impacts of Land Use Changes on Net Primary Productivity in Urban Agglomerations under Multi-Scenarios Simulation. Remote Sensing. 2022;14(7). doi: 10.3390/rs14071755. PubMed PMID: WOS:000781795000001.

39. Liang X, Guan QF, Clarke KC, Liu SS, Wang BY, Yao Y. Understanding the drivers of sustainable land expansion using a patch-generating land use simulation (PLUS) model: A case study in Wuhan, China. COMPUT ENVIRON URBAN. 2021;85. doi: 10.1016/j.compenvurbsys.2020.101569. PubMed PMID: WOS:000596814400016.

40. Wang S, Chen Y, Jin H, Li Y. Ecological management zoning based on the causation between ecological risk and ecosystem services in the Gaoligong Mountain. ECOL INDIC. 2024;167:112673. doi: https://doi.org/10.1016/j.ecolind.2024.112673.

41. Liu H, Tang D. Ecological zoning and ecosystem management based on landscape ecological risk and ecosystem services: A case study in the Wuling Mountain Area. ECOL INDIC. 2024;166:112421. doi: https://doi.org/10.1016/j.ecolind.2024.112421.

42. Shen Q, Gao G, Duan Y, Chen L. Long-term continuous changes of vegetation cover in desert oasis

of a hyper-arid endorheic basin with LandTrendr algorithm. ECOL INDIC. 2024;166:112418. doi: https://doi.org/10.1016/j.ecolind.2024.112418.

43. Chouari W. Assessment of vegetation cover changes and the contributing factors in the Al-Ahsa Oasis using Normalized Difference Vegetation Index (NDVI). REG SUSTAIN. 2024;5(1):100111. doi: https://doi.org/10.1016/j.regsus.2024.03.005.

44. Xue W, Yan L, Gui-Qing X. Response of two dominant woody species to groundwater depth at the transition zone between desert and oasis. ECOL INDIC. 2024;166:112278. doi: https://doi.org/10.1016/j.ecolind.2024.112278.

45. Du L, Gong F, Zeng Y, Ma L, Qiao C, Wu H. Carbon use efficiency of terrestrial ecosystems in desert/grassland biome transition zone: A case in Ningxia province, northwest China. ECOL INDIC. 2021;120:106971. doi: https://doi.org/10.1016/j.ecolind.2020.106971.

46. Chen X, Zhu B, Liu Y, Li T. Ecological and risk networks: Modeling positive versus negative ecological linkages. ECOL INDIC. 2024;166:112362. doi: https://doi.org/10.1016/j.ecolind.2024.112362.

47. Mansour S, Alahmadi M, Atkinson PM, Dewan A. Forecasting of Built-Up Land Expansion in a Desert Urban Environment. Remote Sensing. 2022;14(9). doi: 10.3390/rs14092037. PubMed PMID: WOS:000794805200001.

48. Yao LT, Zhang XB, Luo J, Li XH. Identification of Ecological Management Zoning on Arid Region from the Perspective of Risk Assessment. Sustainability. 2023;15(11). doi: 10.3390/su15119046. PubMed PMID: WOS:001004788700001.

49. Kang L, Yang X, Gao X, Zhang J, Zhou J, Hu Y, et al. Landscape ecological risk evaluation and prediction under a wetland conservation scenario in the Sanjiang Plain based on land use/cover change. ECOL INDIC. 2024;162:112053. doi: https://doi.org/10.1016/j.ecolind.2024.112053.

50. Chen W, Zhao R, Lu H. Response of ecological environment quality to land use transition based on dryland oasis ecological index (DOEI) in dryland: A case study of oasis concentration area in Middle Heihe River, China. ECOL INDIC. 2024;165:112214. doi: https://doi.org/10.1016/j.ecolind.2024.112214.

51. Cao Y, Zhang M, Zhang Z, Liu L, Gao Y, Zhang X, et al. The impact of land-use change on the ecological environment quality from the perspective of production-living-ecological space: A case study of the northern slope of Tianshan Mountains. ECOL INDIC. 2024;83:102795. doi: https://doi.org/10.1016/j.ecoinf.2024.102795.

52. Wang X-d, Xu H-j, Pan Y-x, Fu G-q, Chen T, Qi X-l, et al. Vegetation responses to ecological water delivery and prolonged droughts in an artificial desert oasis, Northwestern China. ECOL INDIC. 2023;77:102284. doi: https://doi.org/10.1016/j.ecoinf.2023.102284.

53. Zhou Q, Chen W, Wang H, Wang D. Spatiotemporal evolution and driving factors analysis of fractional vegetation coverage in the arid region of northwest China. SCI TOTAL ENVIRON. 2024;954:176271. doi: https://doi.org/10.1016/j.scitotenv.2024.176271.

54. Zhao Y, Wang Y, Zhou J, Qi L, Zhang P. Spatiotemporal variation and controlling factors of dried soil layers in a semi-humid catchment and relevant land use management implications. CATENA. 2024;240:107973. doi: https://doi.org/10.1016/j.catena.2024.107973.

55. Ding RC, Li YC, Zhao CZ, Chen JP, Zhu WH. Scenario Simulation and Driving Force Analysis of Ecosystem Service Values Based on Land Use/Cover in the Tumen River Basin, China. Sustainability. 2024;16(1). doi: 10.3390/su16010399. PubMed PMID: WOS:001140386700001.

56. Dunford R, Harrison PA, Jäger J, Rounsevell MDA, Tinch R. Exploring climate change

vulnerability across sectors and scenarios using indicators of impacts and coping capacity. CLIMATIC CHANGE. 2015;128(3-4):339-54. doi: 10.1007/s10584-014-1162-8. PubMed PMID: WOS:000348802400013.

57. Kanellopoulos A, Reidsma P, Wolf J, van Ittersum MK. Assessing climate change and associated socio-economic scenarios for arable farming in the Netherlands: An application of benchmarking and bio-economic farm modelling. European Journal of Agronomy. 2014;52:69-80. doi: 10.1016/j.eja.2013.10.003. PubMed PMID: WOS:000328924600007.

Click here to access/download
Supporting Information
Supplementary data.docx
