# Supplementary material for: Landscape structure evolution and ecological risk evaluation of oasis desert cities: A case study of Tiemenguan city
Source: PLoS One. 2025 May 7;20(5):e0321762. doi: 10.1371/journal.pone.0321762 (PMC12057882; doi:10.1371/journal.pone.0321762)
Supplement: Attachment — Submitted filename: Response to Reviewers 2.docx [file pone.0321762.s006.docx]

**Detailed Response to Reviewers**

**Dear Reviewers：**

Thank you very much for allowing us to revise and resubmit our manuscript entitled “**Landscape Structure Evolution and Ecological Risk Evaluation of Oasis Desert Cities: A Case study of Tiemenguan City**” (Manuscript Number: PONE-D-24-50511). We greatly appreciate the editor and reviewers for your constructive and positive comments and suggestions on our manuscript.

We have carefully studied the reviewers’ comments, made revisions to the revised manuscript, and did our best to improve our manuscript according to the comments. Below are the replies to the reviewer’s comments. Please find the revised version attached, which we would like to resubmit for your kind consideration.

**Reviewer #2**

1. **The abstract is correct, but more information about how the parameters were done could be included.**

**Responses:**

**(1) Thanks very much for the reviewer's kind suggestion to improve this paper.** **We have added details of the parameter settings in the summary section. The modifications are displayed as follows (lines 23 - 28):**

**The normalized difference vegetation index (NDVI) thresholds were NDVI ≥ 30% for oases, 10% < NDVI < 30% for transition zones, and NDVI ≤ 10% for desert areas. Under government control, transitions from cropland, woodland, and grassland to built-up and unused land decreased by 20%, whereas conversions from unused land to cropland, woodland, and grassland increased by 30%.**

**(2) Thanks very much for the reviewer's kind suggestion to improve this paper.** **We have upsized and reordered Figure 1 and also submitted the original of the figure.**

**Fig 1. Research framework. Notes: LUTD, LDI, LFI, and LLD stand for Land Use Type Data, Landscape Disturbance Index, Landscape Fragility Index, and Landscape Loss Index, respectively.**

**(3) Thanks very much for the reviewer's kind suggestion to improve this paper.** **It was our oversight that caused this error, having deleted “of” in “Area statistics for OF oases...”**

2. **Line 414, delete ".." in the phrase: "the evolution of the OLS [50, 51]. ."**

**Responses:**

**Thanks very much for the reviewer's kind suggestion to improve this paper. It was our oversight that caused this error, having deleted “.” in “the evolution of the OLS [50, 51]. .”**

3. **In the "Discussion" section, it would be appropriate to relate results and discussions directly with previous related studies.**

**Responses:**

**Thanks very much for the reviewer's kind suggestion to improve this paper. We have added the results of previous studies in the “Discussion” section that are directly linked to this studies. The modifications are displayed as follows：**

**This result is similar to the findings of Wang et al., who concluded that the ecological risks ranged from low to high in core areas (where human activities were concentrated), buffer zones, and native areas (where anthropogenic disturbances were low) [43].**

**Wang et al. and Liu and Tang concluded that differences in ecological management methods and the degree of disturbance affected ecological risk. They observed an increase in the oasis area as management measures improved [44, 45].**

**Zhang et al. also observed a circular landscape pattern in the oasis region and significant differences in the landscape structure, which were related to the human activities and ecosystem resilience [49].**

**Gan et al. and Chen et al. also observed small effects of elevation and slope on ecological risk [33, 50].**

**Zhou et al. demonstrated that prolonged drought resulted in a decline in vegetation vitality in the oasis, affecting the region’s ecological stability. Effective ecological management stabilizes vegetation growth by regulating the water environment [60].**

**References：**

**33.	Gan L, Halik Ü, Shi L, Welp M. Multi-scenario dynamic prediction of ecological risk assessment in an arid area of northwest China. Ecol Indic. 2023;154: 110727.**

**43.	Wang H, Liu X, Zhao C, Chang Y, Liu Y, Zang F. Spatial-temporal pattern analysis of landscape ecological risk assessment based on land use/land cover change in Baishuijiang National nature reserve in Gansu Province, China. Ecol Indic. 2021;124: 107454.**

**44.	Wang S, Chen Y, Jin H, Li Y. Ecological management zoning based on the causation between ecological risk and ecosystem services in the Gaoligong Mountain. Ecol Indic. 2024;167: 112673.**

**45.	Liu H, Tang D. Ecological zoning and ecosystem management based on landscape ecological risk and ecosystem services: A case study in the Wuling Mountain Area. Ecol Indic. 2024;166: 112421.**

**49.	Zhang W, Ma L, Li H, Wang X. Trends in the future evolution of rural settlements in oasis-desert areas under water use simulation scenarios: Take the Hexi Corridor region of China as an example. Landscape and Urban Planning. 2024;248: 105110.**

**50.	Chen X, Zhu B, Liu Y, Li T. Ecological and risk networks: Modeling positive versus negative ecological linkages. Ecol Indic. 2024;166: 112362.**

**60.	Zhou Q, Chen W, Wang H, Wang D. Spatiotemporal evolution and driving factors analysis of fractional vegetation coverage in the arid region of northwest China. Sci Total Environ. 2024;954: 176271.**

4. **The "Conclusion" section needs to be improved. It is closer to a synthesis of the results than to conclusions.**

**Responses:**

**Thanks very much for the reviewer's kind suggestion to improve this paper. We have rewritten the "Conclusion" section to meet the need for a synthesis of the results throughout the text. The modifications are displayed as follows (lines 616 - 636):**

**(1) The oasis zone expanded significantly during the 30-year study period, increasing from 175.5 km²in 1990 to 345.3 km²in 2020, whereas the desert zone shrank by 37.9% from 257.8 km² to 160.0 km². The ERI thresholds were 0.08–0.085 for the oasis-transition zone and 0.111–0.118 for the transition-desert zone, highlighting the transitional zone's critical role in ecological stability.**

**(2) Socioeconomic factors had a dominant influence on the ERI, with infrastructure expansion and population density exerting the most significant effects (the relative importance values exceeded 0.21). In contrast, the influence of natural factors like precipitation and temperature decreased over time. The highest ERI decrease was from 0.182 in 2000 to 0.171 in 2020, reflecting effective ecological management measures.**

**(3) Under the government-controlled scenario, ecological risk is projected to decline by 2030, with low-risk areas increasing from 36.3% under the natural growth scenario to 39.6% and high-risk areas decreasing from 4.7% to 4.5%. The transition zone area is expected to grow significantly to 82.5 km², whereas the oasis zone is predicted to expand to 375.3 km², a slower growth compared to the natural growth scenario (382.6 km²), demonstrating the efficacy of sustainable land-use policies.**

**This research underscores the importance of balancing socioeconomic development with ecological sustainability in arid regions. The findings highlight the need for integrated management approaches that enhance the resilience of oasis desert landscapes. Future studies should perform similar studies in diverse geographical and socioeconomic settings to promote sustainable urbanization in fragile ecosystems.**

5. **The "Conclusion" section needs to be improved. It is closer to a synthesis of the results than to conclusions.**

**Responses:**

**Thanks very much for the reviewer's kind suggestion to improve this paper. We have revised the reference format according to the journal's formatting guidelines requirements.**
